# Acceptability of telephone-cardiopulmonary resuscitation (T-CPR) practice in a resource-limited country- a cross-sectional study

**DOI:** 10.1186/s12873-022-00690-w

**Published:** 2022-08-02

**Authors:** Fareed Ahmed, Uzma Rahim Khan, Salman Muhammad Soomar, Ahmed Raheem, Rubaba Naeem, Abid Naveed, Junaid Abdul Razzak, Nadeem Ullah Khan

**Affiliations:** 1 Department of Emergency Medicine, Aga Khan University, Karachi, Pakistan; 2 Sindh Rescue & Medical Services, Karachi, Pakistan; 3 Emergency Medicine, Weill Cornell Medicine, New York City, USA

**Keywords:** Emergency, Telephone Cardiopulmonary Resuscitation, Cardiac Arrest, Survival, Bystander

## Abstract

**Background:**

T-CPR has been shown to increase bystander CPR rates dramatically and is associated with improved patient survival.

**Objective:**

To evaluate the acceptability of T-CPR by the bystanders and identify baseline quality measures of T-CPR in Karachi, Pakistan.

**Methods:**

A cross-sectional study was conducted from January to December 2018 at the Aman foundation command and control center. Data was collected from audiotaped phone calls of patients who required assistance from the Aman ambulance and on whom the EMS telecommunicator recognized the need for CPR and provided instructions. Information was recorded using a structured questionnaire on demographics, the status of the patient, and different time variables involved in CPR performance. A One-way ANOVA was used to compare different time variables with recommended AHA guidelines. *P*-value ≤ 0.05 was considered significant.

**Results:**

There were 481 audiotaped calls in which CPR instruction was given, listened to, and recorded data. Out of which in 459(95.4%) of cases CPR was attempted Majority of the patients were males (*n* = 278; 57.8%) and most had witnessed cardiac arrest (*n* = 470; 97.7%) at home (*n* = 430; 89.3%). The mean time to recognize the need for CPR by an EMS telecommunicator was 4:59 ± 1:59(min), while the mean time to start CPR instruction by a bystander was 5:28 ± 2:24(min). The mean time to start chest compression was 6:04 ± 1:52(min.).

**Conclusion:**

Our results show the high acceptability of T-CPR by bystanders. We also found considerable delays in recognizing cardiac arrest and initiation of CPR by telecommunicators. Further training of telecommunicators could reduce these delays.

## Introduction

It has been widely reported and accepted that rapid initiation of cardiopulmonary resuscitation (CPR) benefits the overall survival of patients with out-of-hospital cardiac arrest (OHCA).[1–3]. To get a successful resuscitation of OHCA victims, the American Heart Association (AHA) has endorsed the “Chain of Survival” as a framework [4]. The first link of the “Chain” is immediate recognition of cardiac arrest and early bystander cardiopulmonary resuscitation [BCPR]) of good quality with minimum interruption are strongly associated with improved survival in such patients.[5, 6]. The time between an emergency call and the arrival of emergency medical services (EMS) is one of the most critical time intervals for the outcomes of patients with OHCA. An attempt of cardiopulmonary resuscitation administered by a family member, a friend, or a nearby person in the community before emergency medical services, is called bystander cardiopulmonary resuscitation (BCPR). [7, 8]. It has been observed that BCPR is provided in only one-third to one-half of cases in most communities, despite this intervention being more than double the chance of survival in OHCA patients. [9]. However, the quality of BCPR is often poor, and overall, the number of patients receiving CPR is still very low. [10]

Telephone CPR (T-CPR) is a technique that provides instructions to callers of suspected OHCA cases about how to deliver compression and ventilation. Since this intervention holds enormous potential to increase bystander response to performing CPR and thus survival from cardiac arrest, T-CPR has been recognized as an integral component of an emergency medical system response to OHCA.[11]. In T-CPR, instructions were given verbally by telephone to a suspected case of cardiac arrest victim to increase the number of patients receiving CPR and improve the quality of CPR delivered [12]. However, bystanders often face multiple barriers to performing CPR, causing a delay in CPR initiation, and compromising the quality of CPR being provided[13]. Nevertheless, a study supported the positive impact of this intervention with a continuous quality improvement project as reported by the Ishikawa Medical Control Council in 2007, where they found a substantial decrease in the incidence of failed telephone CPR due to human factors with a drastically increased in bystander CPR through this intervention[14].

In India, a study was conducted to determine the acceptability of T-CPR. They found that in 599 cases of out-of-hospital cardiac arrest victims, instructions to perform CPR were given to the bystanders, and in the majority of cases, 482 (80%) CPR was not performed. In only, 117, (20%) cases CPR was attempted[15]. There is limited data on the use of TCPR in developing countries like Pakistan, especially little is known about the acceptability of TCPR amongst bystanders in our setting. The primary objective of this study was to evaluate the acceptability of T-CPR by the bystanders, and the secondary objective was to identify baseline quality measures of T-CPR in Karachi, Pakistan.

### Operational definition

#### Acceptability of T-CPR

Acceptability of T-CPR was defined as performing CPR by bystander upon instructions provided by a telecommunicator [16] .

## Methods

Study design and setting: We conducted a cross-sectional study and included all adults who met the criteria of Medical Priority Dispatch System (MDPS) code 9 & 31 and MPDS priority system code 3 [17] from January 2018 to December 2018.

### Study setting

The study was conducted in Karachi, the largest city in Pakistan, with Several private, not-for-profit ambulance services serving the city. In Karachi, the public does not have one standard telephone number for a medical emergency and must call specific ambulance services. One of the major ambulance service providers is Aman Ambulance (more recently called Sindh Emergency Health and Rescue services), which provides advanced ambulance service with trained nurse paramedics. The ambulance call center has trained emergency medical telecommunicators who provide T-CPR. As far as we know, Aman Ambulance is the only service in Pakistan providing T-CPR.

### Study procedure and data collection

We collaborated with Aman Ambulance service and obtained recordings of all telephone calls on which T-CPR instruction was provided. Hands-only CPR was performed. The research assistant (RA) trained for this study collected data from audio calls. We developed a questionnaire to collect data on age, gender, time to recognize the need for CPR, time to CPR instructions, time to initiation of BCPR, time of ambulance arrival, and barriers to performing CPR. Minor modifications were made to the structure of some questions after pre-testing with 40 participants. We included those patients on whom T-CPR instructions were provided by telecommunicator. To protect the privacy of the study participants, each person was given a unique study ID which was noted on top of the survey questionnaire.

### Analysis

Mean and Standard Deviation (SD) were calculated for continuous variables such as age, time to recognize the need for CPR, time to start CPR instruction, and time to start chest compression. Normality was checked through Shapiro–wilk test. Frequency and percentages were calculated for qualitative variables such as gender, scene of the event, and place of the event. We compared our results with the American Heart Association guidelines for T-CPR since they are applied in Pakistan. AHA divides its recommendation into the high-performance target and the minimum expected target. A One-way ANOVA was used to compare different time variables with recommended standards.

## Results

A total of 481 audiotaped calls in which CPR instruction was reviewed. Most OHCA occurred at home (89.3%; *n* = 430). Most of the bystanders started CPR when instructed by the telecommunicators. (95.4%; *n* = 459). 57.8% (*n* = 278) of patients on which CPR was performed were male.(See Table 1).

**Table 1:** General descriptive variables of the study

**Variables**	Mean ± SD/%
Age	64.45 ± 15.83
Sex	
Male	278 (57.8%)
Female	203 (42.2%)
Witnessed arrest	
Yes	470(97.7%)
No	11(2.3%)
Place of Event	
Home Outside Home	430(89.3%) 51(10.7%)

The mean time to recognize the need for CPR by a telecommunicator was 4:59 ± 1:59(minutes), while the mean time to start CPR instruction by a bystander was 5:28 ± 2:24(min). The mean time to start chest compression was 6:04 ± 1:52(min.) (See Table 2).

**Table 2:** Measures of time for T-CPR

**Time measures**	Mean ± SD (Mins: Sec)
Mean time to recognize the need for CPR	4:59 ± 1:59
Mean time to start CPR instruction	5:28 ± 2:24
Mean time to start chest compression	6:04 ± 1:52

Compared to the AHA timeline recommendations, they have divided into the high-performance system and minimal acceptable. In 3 (0.6%) of cases, the time to recognize the need for CPR was less than a minute, and in 28(5.8%) of patients, it was less than 2 min, while 440(93.5%) cases were beyond this timeline. In only 1(0.2%) of cases, CPR instruction was started within a minute, and in 14(3%) of patients, it was within 2 min, but in 453(96.8%) of cases, it was beyond that timeline. From time to the first compression, none met a high-performance system, and in 40(8.7%) cases, it was started within 3 min, meeting a minimal acceptable limit, while the rest was out of range. (See Table 3).

**Table 3:** Comparison of different time variants with recommended AHA guidelines

**Variables**	**Mean + SD**	**Range** **(min. – max.)**	**Performance target**			**p-value**
			**High performance** **target**	**Minimal** **acceptable** **target**	**Below minimum target**	
**Time to recognize the need for CPR?** **(min:sec)**	4:59 (1:59)	(1:20 – 14:40)	< 1 min 3(0.6%)	< 2 min 28(5.8%)	440(93.5%)	0.532*
**Time to CPR instructions** **started? (min:sec)**	5:28 (2:24)	(1:40 – 40:30)	< 1 min 1(0.2%)	< 2 min 14(3%)	453(96.8%)	0.761*
**Time to the first compression** **(min:sec)**	6:04 (1:52)	(2:15 – 16:00)	< 2 min 0(0%)	< 3 min 40(8.7%)	418(91.3%)	0.977*

^*^One-way ANOVA

## Discussion

We aimed to assess the acceptability of T-CPR by the bystanders and measure baseline quality indicators for the T-CPR in Karachi, Pakistan. The study result showed that in more than ninety percent of cases, CPR was attempted after instructions provided on the phone.

A study reported that the most predominant barriers against telecommunicator-assisted CPR are emotional factors, including panic and hysteria [18]. McCormack AP also reported that willingness to perform CPR by a bystander might be affected by the patient's physical characteristics and its surrounding, such as vomiting were observed in 59% of cases had a negative impact on performing CPR [19]. Other studies also observed that, in a public place, an AED pad application or receiving bystander CPR for female OHCA patients was less difficult, especially in their reproductive age group.[20–22]. This could be possibly due to the cultural and social differences in different parts of the world.

We found a high level of willingness to perform CPR. This could be due to victims being known to the bystanders since most OHCA occurred at home. In a study by Birkun et al., about 79% of bystanders were willing to perform CPR on unknown people, and 91% were willing to attempt CPR on their friend or relative [23]. However, a study conducted in India in 2017 by Jyothi showed that 80% of the bystanders did not do CPR despite the telephonic instructions being given. [15]

We also noticed in our study that the majority of the bystander who performed CPR were females. This could be because women were more likely to be at home than their male partners. Although we did not collect the data on whether those bystanders who perform CPR had previous knowledge of it or had any formal training to perform CPR, it has been proven that if a person has received CPR training within the last five years or they have trained in their high school education will more likely willing to perform bystander CPR [24, 25].

We also assessed the baseline quality measures of CPR in the current situation and compared them with the AHA recommendations. We found significant delays in time (5 min) to recognize the need for CPR, with more than 90% being delayed beyond the range recommended by the AHA.,. In a study by Stangenes et al., the median time interval for recognizing T-CPR was 47 s. for valid medical complaints, and it took 100 s for false medical complaints[26]. Another study by Demi et al. reported a median time to recognize the need for CPR of 60 s [27]. Possible reasons for the delay in recognition of the need for CPR could be since most of them out of hospital cardiac arrest (OHCA) happen at home, allowing only a limited number of people to respond. Also, there is a lack of awareness in our world that doesn’t enable the bystanders to recognize the situation and the need for CPR immediately. [5]

The mean time to start the CPR instructions was also more than 5 min, which should ideally be less than 2 min; about 96.8% of cases were out of range. Similar findings were found in a study by Lewis et al. where they found a delay in CPR instruction by more than five seconds in 92.9% of cases. A proportion of the delays could be attributed to the telecommunicator asking superfluous incident and medical history questions after establishing that the patient was unconscious and not breathing[28]. The high mean time for the telecommunicators to start giving the instructions could be because OHCA is usually a panicky situation, [8] the family members or other bystanders cannot communicate effectively, let alone the delayed recognition, as a layperson. Our study also shows the delay at the telecommunicator’s side.

The mean time to start the first chest compression took 6 min, twice the recommended range (minimal acceptable < 3 min). A study by Hardeland in a developed country showed differences in the timings from that of our population. Their median time (mm: ss) to first chest compression was 02:35 (Copenhagen), 03:50 (Stockholm), and 02:58 (Oslo). [29].

The importance of this intervention was also highlighted in a study by Eisenberg et al., where they found that OHCA survival to discharge jumped from 6 to 21%, and the rates of bystander CPR were also increased from 32 to 55% after they implemented a dispatch-assisted CPR program in their EMS. Hence due to early recognition and prompt actions of their telecommunicators. King County report one of the world's highest cardiac arrest survival rates [30] A Dutch study CPR was not associated with ROSC or 30-day survival. Dispatcher-assisted CPR was especially beneficial for initiating bystander CPR in residential areas [31].

Our study highlights several areas of potential improvement. First, improved training and quality can considerably improve the time to recognize the need for CPR and the quality of instructions. Secondly, effective and timely CPR can be achieved when a considerable percentage of the population is trained in CPR through a national program targeted at the formal and informal schooling system. The training should involve both men and women in society to improve effective BCPR at home and outside.

Limited EMS facilities in the country, lack of centralized EMS, and their routine T-CPR training are concerns that need to be addressed. [15, 32]. In a study by Hasan et al., a DACPR implementation positively impacted the Kuwait EMS system; after DACPR implementation, the OHCA recognition rate increased from 2 to 12.9%, CPR instruction rate increased from 0 to 10.4%. [33]. They also had a similar conclusion as that of our study that the lack of knowledge of CPR skills and training among bystanders in the community is why most OHCA patients in India do not get appropriate and timely CPR. [15].

### Strength and Limitations

This is the first-ever study in Pakistan evaluating the acceptability of T-CPR in an LMIC. There are a few limitations to this study. First, this was a single EMS system study from one city in Pakistan and did not represent all EMS systems and providers in the country. Most EMS providers in Pakistan don’t offer TCPR. Secondly, we used one-time data based on the recordings as this was a cross-sectional study and did not have a follow-up. Thirdly, we did not collect information on the patient outcomes. Fourthly, we did not have any information on patients with cardiac arrests on which no DA CPR instructions were given; thus, we don’t know the percent of patients with cardiac arrest who received DA-CPR.

## Conclusion

Our results show the high acceptability of T-CPR by bystanders. We also found considerable delays in recognizing cardiac arrest and initiation of CPR by telecommunicators. Further training of telecommunicators could reduce these delays.

## Data Availability

The data will be available at the reasonable request to Dr. Fareed Ahmed, the corresponding author at fareed.ahmed@aku.edu.

## Abbreviations

TA-CPR: Telecommunicator-Assisted Cardiopulmonary Resuscitation
EMS: Emergency Management System
OHCA: Out of Hospital Cardiac Arrest
RA: Research Assistant
T-CPR: Telephone Cardiopulmonary Resuscitation
